# Dietary ferulic acid improves growth performance of broilers via enhanced intestinal antioxidant capacity and barrier function

**DOI:** 10.5713/ab.23.0487

**Published:** 2024-04-26

**Authors:** Yaodong Hu, Shi Tang, Wei Zhao, Silu Wang, Caiyun Sun, Binlong Chen, Yuxing Zhu

**Affiliations:** 1 College of Animal Science, Xichang University, Xichang, 615000, China

**Keywords:** Antioxidant Capacity, Ferulic Acid, Growth Performance, Intestinal Integrity

## Abstract

**Objective:**

In this study, the effects of dietary ferulic acid (FA) on the growth traits, antioxidant capacity, and intestinal barrier function of broilers were investigated.

**Methods:**

In total, 192 male Arbor Acres broilers were randomly allocated to one of three dietary groups (8 replicates of 8 birds each): control (CON) group (basal diet), FA100 group (basal diet + 100 mg/kg FA), or FA200 group (basal diet + 200 mg/kg FA). The duration of the feeding trial was 42 days.

**Results:**

Higher average daily gain (ADG) and lower feed to gain (F/G) ratio during day 0 to day 21 were found in the FA100 and FA200 groups, while higher ADG and lower F/G during day 21 to day 42 were only found in FA200 group, compared to the CON group (p<0.05). Serum levels of malondialdehyde and diamine oxidase on day 21 were lower in the FA100 and FA200 groups and those on day 42 were lower in the FA200 group, while glutathione peroxidase level in the FA100 and FA200 groups on day 21 and that in the FA200 group on day 42 were increased (p<0.05). On day 21, jejunal glutathione synthetase (*GSS*) expression was upregulated in the FA200 group (p<0.05), while jejunal and ileal expression of nuclear factor erythroid 2-related factor 2 (*NRF2*) and *Occludin* as well as ileal expression of glutathione peroxidase 1 (*GPX1*) and zonula occludens 1 (*ZO1*) were increased in the FA100 and FA200 groups compared to the CON group (p<0.05). On day 42, mRNA expression of *GSS*, *NRF2*, *SOD1*, and *GPX1* in the jejunum and ileum as well as *Claudin2* in the jejunum and *Occludin* in the ileum were increased in the FA200 group (p<0.05).

**Conclusion:**

Dietary FA addition could improve the growth performance, antioxidant capacity, and gut integrity of broilers. The current findings provided evidence that the adoption of FA can be a nutrition intervention measure to achieve high-efficient broiler production for poultry farmers.

## INTRODUCTION

Genetic selection to improve the growth rate and feed efficiency of broilers has led to increased susceptibility to oxidative stress (OS) and redox imbalances [1]. Various oxidative factors such as high environmental temperatures, dietary oxidized lipids, pathogens, toxins, and hazardous materials could disrupt redox balance and induce OS and oxidative damage [2,3]. Multiple stressors are known to induce oxidative damage to DNA, proteins, and lipids [2,4], resulting in growth inhibition, disruption of cellular antioxidation, destruction of intestinal integrity, dysbiosis of the gut microbiota, and diminished meat quality [5,6]. Intestinal barrier plays key roles in maintaining intestinal homeostasis [7]. Inflammation and oxidative damage can severely destroy the intestinal integrity [8]. Enhancing intestinal antioxidant capacity and barrier function were important for broilers efficient and healthy production.

Dietary supplementation with antioxidants can effectively alleviate the harmful effects of OS in livestock and poultry [9]. Ferulic acid (FA) is a type of organic compound belonging to the family of hydroxycinnamic acids and commonly derived from the seeds, leaves, and fruits of various plant species [10]. The FA is reported to exhibit antioxidant, anti-inflammatory, anticancer, antiaging, and neuroprotective effects [11]. Previous studies have demonstrated that FA effectively repressed cellular lipid peroxidation by clearing free radicals [12]. Owing to strong antioxidant activities, FA is often applied as an additive to animal feed [13]. Dietary FA has been shown to benefit the growth performance (GP) and health of pigs and rodents via regulation of cellular antioxidant defense [12,14,15]. However, relatively few studies have investigated the influence of dietary FA in poultry. Previous studies have shown that dietary supplementation with FA-rich propolis improved the weight gain and feed conversion efficiency of laying hens and meat ducks [16,17]. A recent study indicated that FA enhanced the GP of broilers by improving digestive enzyme activities and restoring hepatic redox status [18]. Moreover, ferulic supplementation has been demonstrated to improve intestinal heath of LPS-challenged broilers [17]. Nonetheless, the effects of different dietary FA levels on the intestinal health of broilers remain unknown, as the single dose of FA was applied in all the previous studies. Therefore, the aim of the present study was to assess the effects of dietary FA levels on the intestinal redox balance, intestinal integrity, and intestinal immune function of broilers. The results of this study provide useful references for the application of FA in broiler feed.

## MATERIALS AND METHODS

### Study approval

The study protocol was approved by the Institutional Animal Care and Use Committee of Xichang University (approval no.XC20220105) and conducted in accordance with the Guide for the Care and Use of Laboratory Animals.

### Animal experiments

In total, 192 male Arbor Acres broilers (age, 1 day; mean body weight, 43.80±0.81 g) were randomly allocated to one of three dietary groups (8 replicates of 8 birds each): control (CON) group (basal diet without FA), FA100 group (basal diet + 100 mg/kg FA), or FA200 group (basal diet + 200 mg/kg FA). The duration of the feeding trial was 42 days. The corn/soybean-based basal diet was formulated to meet the nutritional requirements for broilers as recommended by the National Research Council (1994). The ingredients and nutrient levels of the basal diet are listed in Table 1. All diets were offered to broilers in mash form during the starting phase (first 3 weeks) and in pellet form during the growing phase (second 3 weeks). The dosage of FA in this study was based on previous studies, which found that dietary FA at 80 ppm or 100 ppm enhanced the GP of broilers [16,17]. The FA product (purity >98%), extracted from the stem and root of Ferula asafoetida Linn., was purchased from Yuanye Bio-Technology Co., Ltd (Shanghai, China). Broiler chickens were raised in wire cages with plastic floors and water nipples (125 cm [length]×75 cm [width]×50 cm [height]) in a temperature-controlled room with *ad libitum* access to feed and water during the entire experimental period. Management of chicks was performed in accordance with established guidelines for raising of Arbor Acres broilers.

### Sample collection

On days 21 and 42, after fasting for 12 h, blood samples were collected from the wing vein of one bird from each replicate and centrifuged at 3,500 rpm for 10 min at 4°C. The upper serum portion was transferred to a clean vial and stored at −20°C until analyzed. Afterward, the birds were euthanized by cervical dislocation and the duodenum, jejunum, and ileum were collected. The mucosal scrapings and tissues of each section of the intestine were stored at −80°C until analyzed.

### Growth performance

On days 21 and 42, after feed withdrawal for 12 h, the total body weight of birds in each cage was measured to calculate the average daily gain (ADG) at day 0 to day 21, day 22 to day 42, and day 0 to day 42. In addition, the average daily feed intake (ADFI) was recorded for each cage. The feed to gain (F/G) ratio at day 0 to day 21, day 22 to day 42, and day 0 to day 42 was calculated as ADFI/ADG.

### Measurements of serum biochemical indices

Serum levels of D-lactic acid were measured using a commercial kit purchased from Shanghai Enzyme-linked Biotechnology Co. Ltd. (Shanghai, China), while serum levels of diamine oxidase (DAO), reduced glutathione (GSH), malondialdehyde (MDA), catalase (CAT), superoxide dismutase (SOD), and glutathione peroxidase (GSH-Px) were measured using commercial assay kits purchased from Jiancheng Bioengineering Institute (Nanjing, China).

### Antioxidant assay of intestinal mucosa

The mucosal samples of the duodenum, jejunum, and ileum (0.5 g) were homogenized in phosphate-buffered saline (1:9, w/v). The concentrations of GSH, MDA, CAT, SOD, and GSH-Px in the homogenized mucosa samples were measured with commercial diagnostic kits obtained from Jiancheng Bioengineering Institute.

### Gene expression analysis

Total RNA was isolated from the intestinal specimens using TRIzol reagent (Takara Biotechnology [Dalian] Co., Ltd., Dalian, China) and treated with DNase to remove genomic DNA. The concentration and quality of the extracted RNA samples were assessed with a NanoDrop ND-1000 spectrophotometer (NanoDrop Technologies, LLC, Wilmington, DE, USA) and agarose gel electrophoresis, respectively. Complementary DNA (cDNA) was synthesized from 1 μg of purified RNA using the ImProm-II Reverse Transcription System (Promega Corporation, Madison, WI, USA) and amplified by quantitative polymerase chain reaction (PCR) with KAPA SYBR FAST qPCR Master Mix (Kapa Biosystems, Inc., Wilmington, MA, USA) with the primers listed in Table 2 and a CFX96 Real-Time PCR Detection System (Bio-Rad Laboratories, Hercules, CA, USA). Each 10-μL reaction consisted of 5 μL of 2× master mix, 2 μL of cDNA as a template, 0.5 μL of the forward primer (5 μM), 0.5 μL of the reverse primer (5 μM), and 2 μL of Milli-Q water. Triplicate samples were used for each gene. The qPCR reaction conditions consisted of an initial denaturation step at 95°C for 3 min, followed by 49 cycles at 95°C for 20 s and 60°C for 40 s. Primer specificity was tested by melting curve analysis from 70°C to 90°C with 0.5°C increments every 5 s. The qPCR efficiency was assessed against a standard curve, which was generated by five points of 4-fold serial dilutions of cDNA in each run. The PCR amplification efficiency in this study consistently ranged from 90% to 110%. Relative expression of the target genes was normalized against β-actin as a reference gene using the 2^–ΔΔCt^ method.

### Statistical analyses

Data analyses were performed using SAS 9.4 software (SAS Institute, Inc., Cary, NC, USA). The significance of differences among the treatment groups was determined using one-way analysis of variance followed by the Tukey test. A probability (p) value <0.05 was considered statistically significant. The results are presented as the mean and standard error.

## RESULTS

### Growth performance

The effects of dietary FA on the GP of broilers are shown in Table 3. In groups FA100 and FA200, body weight (p<0.05) increased on day 21, ADG (p<0.05) was increased at day 0 to day 21, and the F/G ratio (p<0.05) was decreased at day 0 to day 21. However, as compared to the CON group, body weight (p<0.05) was increased on day 42, ADG (p<0.05) was increased at day 22 to day 42 and day 0 to day 42, and the F/G ratio (p<0.05) was decreased at day 22 to day 42 and day 0 to day 42 in the FA200 group.

### Intestinal permeability parameters and antioxidant status in serum

The effects of FA on serum levels of biochemical indices of intestinal permeability and antioxidant status are presented in Table 4. On day 21, serum levels of MDA (p<0.05) and DAO (p<0.05) were decreased in groups FA100 and FA200, while serum levels of GSH-Px (p<0.05) were increased and D-lactic acid levels (p<0.05) were decreased in the FA200 group. On day 42, serum activities of T-SOD (p<0.05) and GSH-Px (p<0.05) were increased, while MDA (p<0.05) and DAO (p<0.05) levels were decreased in the FA200 group, but not the FA100 group (p<0.05).

### Antioxidant capacity of the intestinal mucosa

The effects of FA on the antioxidant capacity of the intestinal mucosa of broilers on days 21 and 42 are shown in Table 5 and 6, respectively. On day 21, the GSH contents (p<0.05) were increased and MDA levels (p<0.05) were decreased in the mucosal specimens from the jejunum and ileum of the FA100 and FA200 groups, while the SOD activity (p<0.05) was increased in the ileum as compared to the CON group. On day 42, dietary FA at 200 mg/kg, but not 100 mg/kg, increased total SOD activity (p<0.05) in the jejunum and GSH-Px activity (p<0.05) in the ileum, while the MDA (p<0.05) content was decreased in the ileum.

### Expression patterns of redox-related genes in the intestinal specimens

**Figure 1:**
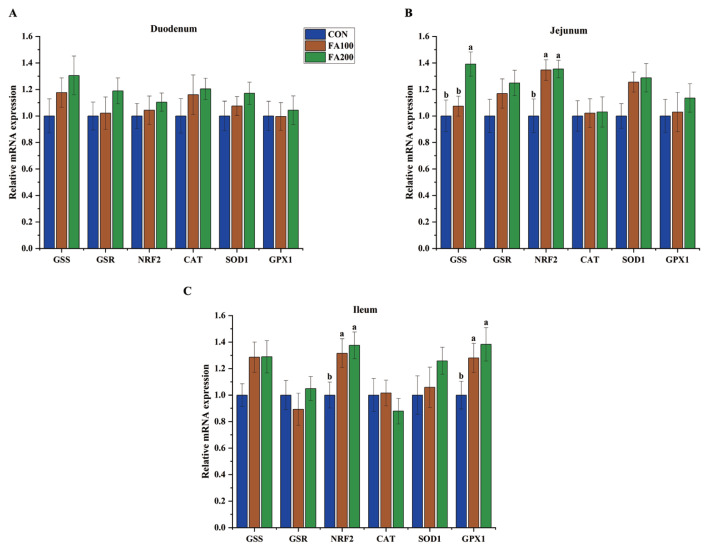
Effects of dietary FA on mRNA expression of redox-related genes in the duodenum (A), jejunum (B), and ileum (C) of broilers on day 21. CON, control group fed the basal diet; FA100, fed the basal diet supplemented with 100 mg/kg FA; FA200, fed the basal diet supplemented with 200 mg/kg FA; FA, ferulic acid; GSS, glutathione synthetase; GSR, glutathione reductase; NRF2, nuclear factor erythroid 2-related factor 2; CAT, catalase; SOD1, superoxide dismutase 1; GPX1, glutathione peroxidase 1. ^a,b^ Different small letters indicate significant differences among treatments (n = 8, p<0.05).

**Figure 2:**
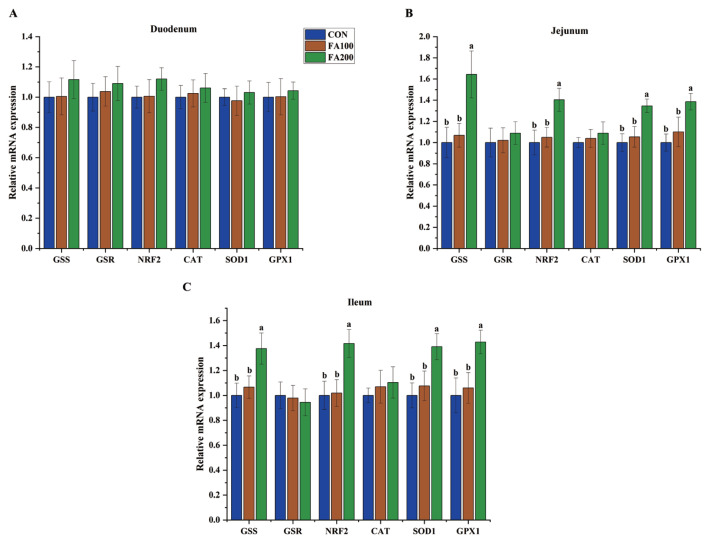
Effects of dietary FA on mRNA expression of redox-related genes in the duodenum (A), jejunum (B), and ileum (C) of broilers on day 42. CON, control group fed the basal diet; FA, ferulic acid; FA100, fed the basal diet supplemented with 100 mg/kg FA; FA200, fed the basal diet supplemented with 200 mg/kg FA; GSS, glutathione synthetase; GSR, glutathione reductase; NRF2, nuclear factor erythroid 2-related factor 2; CAT, catalase; SOD1, superoxide dismutase 1; GPX1, glutathione peroxidase 1. ^a,b^ Different small letters indicate significant differences among treatments (n = 8, p<0.05).

The effects of dietary FA on the expression levels of redox-related genes in the intestinal specimens of broilers on days 21 and 42 are shown in Figures 1 and 2, respectively. On day 21, glutathione synthetase (*GSS*) mRNA expression (p<0.05) was increased in the jejunum in the FA200 group, while mRNA expression of nuclear factor erythroid 2-related factor 2 (*NRF2*) (p<0.05) was increased in the jejunum and ileum of the FA100 and FA200 groups and glutathione peroxidase 1 (*GPX1*) mRNA expression (p<0.05) was increased in the ileum. The mRNA levels of *GSS* (p<0.05), *NRF2* (p<0.05), superoxide dismutase 1 (*SOD1*) (p<0.05), and *GPX1* (p<0.05) were increased in the jejunum and ileum on day 42 in the FA200 group as compared to the CON group.

### Intestinal mRNA expression levels of tight junction proteins

**Figure 3:**
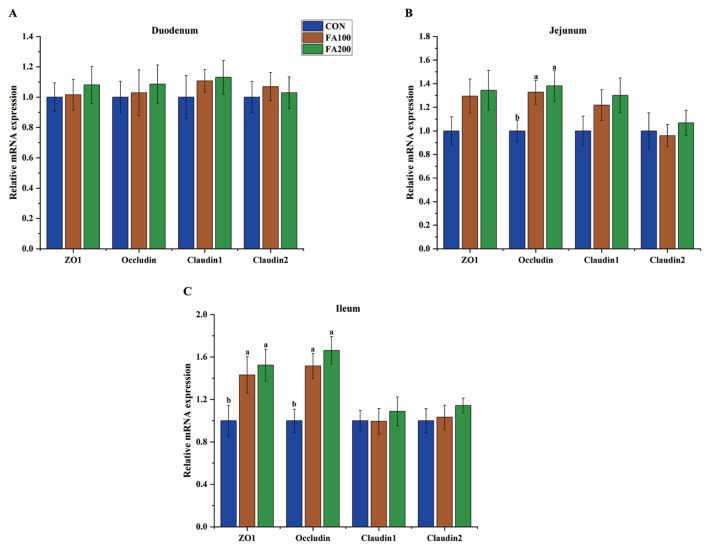
Effects of dietary FA on mRNA expression of TJ proteins in the duodenum (A), jejunum (B), and ileum (C) of broilers on day 21. CON, control group fed the basal diet; FA, ferulic acid; FA100, fed the basal diet supplemented with 100 mg/kg FA; FA200, fed the basal diet supplemented with 200 mg/kg FA; TJ, tight junction; ZO1, zonula occludens 1. ^a,b^ Different small letters indicate significant differences among treatments (n = 8, p<0.05).

**Figure 4:**
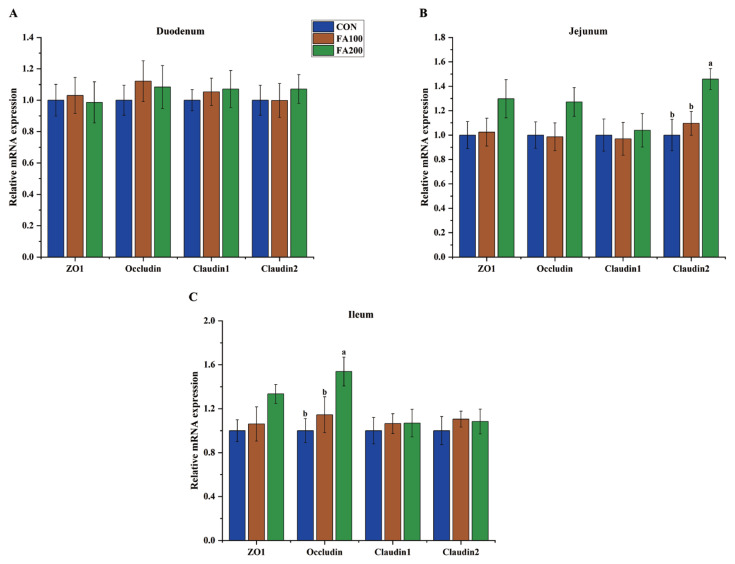
Effects of dietary FA on mRNA expression of TJ proteins in the duodenum (A), jejunum (B), and ileum (C) of broilers on day 42. CON, control group fed the basal diet; FA, ferulic acid; FA100, fed the basal diet supplemented with 100 mg/kg FA; FA200, fed the basal diet supplemented with 200 mg/kg FA; TJ, tight junction; ZO1, zonula occludens 1. ^a,b^ Different small letters indicate significant differences among treatments (n = 8, p<0.05).

The effects of dietary FA on the expression levels of tight junction (TJ) proteins in the small intestine of broilers on days 21 and 42 are shown in Figure 3 and 4, respectively. On day 21, *Occludin* expression (p<0.05) in the jejunum and ileum and zonula occludens 1 (*ZO1*) expression (p<0.05) in the ileum were increased in the FA100 and FA200 groups. On day 42, expression of *Claudin2* (p<0.05) in the jejunum and *Occludin* expression (p<0.05) in the ileum were increased in the FA200 group as compared to the CON group.

## DISCUSSION

Due to the fast growth rate, broilers are especially vulnerable to disturbances to intestinal redox balance and intestinal integrity, which can reportedly be modulated by dietary supplementation [4]. An *in vitro* study showed that dietary FA exhibited strong free radical scavenging ability [19]. Previous *in vivo* studies demonstrated that dietary FA improved the growth rate and antioxidant capacity of piglets under normal conditions and in response to stress [20,21]. However, relatively few studies have explored the effects of dietary FA on broilers. Current evidence indicates that dietary FA increased the growth rate and feed utilization efficiency of broilers during the starter phase, but had no effect on GP of broilers during the grower phase [18]. In the current study, dietary FA at 100 mg/kg improved the ADG and reduced the F/G ratio in broilers in the starter phase, but not the grower phase, while dietary FA at 200 mg/kg improved the ADG and feed conversion efficiency of broilers in the starter and grower phases. These findings indicate that broilers might require higher amounts of FA to promote growth in the grower phase. For broilers production applications, dietary supplementation of FA can increase feed reward, and 1 T diet with 100 mg/kg FA could increase 35.58 ¥ income and diet with 200 mg/kg FA could increase 181.76 ¥ income (98% FA, 330 ¥/kg; broiler, 9.0 ¥/kg body weight). In the growth phase, the redox status of broilers is significantly impacted. A previous study showed that broilers exhibited weaker antioxidant capacity at 42 days of age than 21 days under heat stress conditions [22]. Dietary supplementation with plant extracts is reported to enhance antioxidant capacity, improve intestinal integrity and barrier function, and restore the gut microbiota composition, thereby improving the GP of livestock and poultry [2,21,23]. A previous study showed that dietary FA decreased MDA levels and enhanced GSH-Px activity on day 21, and decreased SOD and GSH-Px activities on day 42 in serum and liver samples [18]. In the present study, dietary FA at 100 and 200 mg/kg increased serum levels of GSH-Px on day 21 and decreased MDA levels. However, on day 42, dietary FA at 200 mg/kg, but not 100 mg/kg, increased serum concentrations of SOD and GSH-Px, but decreased MDA levels. These findings indicate that OS is stronger in the grower phase even under normal conditions [22], and researches showed that antioxidant capacity of young poultry was stronger than that of older [22,24], so age difference might be the reason for the above experiment result.

The intestinal mucosa, which acts as the first line of defense against the external environment, can be easily impaired by various stressors. For example, OS can disrupt redox balance and damage the mucosal barrier of the intestine, resulting in increased intestinal permeability in broilers [2]. Dietary supplementation with antioxidants is a useful strategy to improve OS-induced intestinal defects [13]. The antioxidant properties of FA are beneficial to intestinal redox status and barrier function in piglets, as indicated by the increased CAT activity and reduced MDA content in the jejunum of weaning piglets treated with FA [20,21]. In the present study, dietary FA at 100 and 200 mg/kg increased the GSH content and decreased the MDA content in the jejunum and ileum, and increased SOD activity in the ileum of broilers on day 21, which was consistent with a previous study showing that dietary FA at 80 mg/kg reduced hepatic MDA concentrations of broilers [18]. In addition, dietary FA at 200 mg/kg, but not 100 mg/kg, increased activities of SOD in the jejunum and GSH-Px in ileum, while decreasing MDA concentrations in the ileum. Collectively, these results suggest that FA at 200 mg/kg is a suitable concentration to maintain intestinal redox balance in broilers. More importantly, FA has been reported to activate the NRF2-ARE pathway and associated antioxidant enzymes [25]. Dietary FA at 0.05% and 0.45% has been shown to upregulate mRNA expression of SOD1, GPX1, and NRF2 in the jejunum of weaning piglets [21]. In the present study, dietary FA at 100 and 200 mg/kg enhanced mRNA expression of *NRF2* and *GPX1* in the jejunum and ileum of broilers on day 21, while FA at 200 mg/kg increased mRNA expression of *GSS*, *NRF2*, *SOD1*, and *GPX1* in the jejunum and ileum on day 42. Enhanced activation of antioxidant enzymes and reduced glutathione synthetase activity in the intestine of broilers indicated that FA supplementation had a positive impact on antioxidant capacity and the dosage of FA should be increased with age.

Changes to the intestinal redox state indicate activation of redox-sensitive transcriptional factors, which are associated with disturbances to the integrity of the intestinal mucosa [26]. Previous studies have reported that weaning stress in piglets and OS in broilers caused an imbalance in intestinal redox and impaired intestinal integrity [6,27]. Serum levels of DAO and D-lactic acid are commonly used as biomarkers of intestinal permeability [28]. In the present study, dietary FA at 100 and 200 mg/kg decreased serum levels of DAO in broilers on day 21, while FA at 200 mg/kg decreased the serum content of D-lactic acid on day 21 and DAO serum levels on day 42, indicating that dietary FA enhanced the intestinal integrity of broilers. The results of the present study are in agreement with previous studies, which found that dietary FA decreased the intestinal permeability of piglets, as demonstrated by lower serum concentrations of D-lactic acid and DAO [20,21]. Downregulation or inhibition of TJ protein expression is a common event in response to intestinal redox imbalance and barrier injury [29]. Dietary antioxidants can improve intestinal integrity by upregulating epithelial expression of TJ proteins, which is critical for maintenance of intestinal physical barrier function [30]. Previous studies have demonstrated that dietary FA increased mRNA expression levels of *ZO1*, *ZO2*, *Occludin*, and *Claudin 1* in the jejunum of piglets. In the present study, dietary FA increased mRNA expression levels of *Occludin* and *ZO1* in the jejunum or ileum of broilers on day 21 and enhanced expression of *Occludin* and *Claudin 2* in the jejunum or ileum of broilers on day 42. These findings suggest that the beneficial effects of FA on intestinal permeability might be associated with upregulation of TJ proteins in the intestine.

## CONCLUSION

In summary, dietary FA improved the GP, antioxidant capacity, and intestinal integrity of broilers via enhanced transcription of genes related to redox and TJ proteins. The current findings provided evidences that the adoption of FA can be as nutrition intervention measure to achieve high-efficient broiler production for poultry farmers.

## Figures and Tables

**Table 1:** Ingredient composition and nutrient content of the basal diet

Items	Starter phase (day 0 to 2^1)^	Grower phase (day 22 to 42)
Ingredients (%)		
Maize	56.39	59.65
Soybean meal	36.00	31.83
Soybean oil	3.71	4.69
Limestone	1.27	1.38
Dicalcium phosphate	1.57	1.44
Salt	0.29	0.27
L-Lysine	0.10	0.14
DL-Methionine	0.16	0.15
Choline chloride (50%)	0.26	0.20
Premix^1)^	0.25	0.25
Total	100.00	100.00
Calculated nutrient levels		
Apparent metabolizable energy (MJ/kg)	12.56	12.98
Crude protein	20.5	19.0
Calcium	0.90	0.90
Available phosphorus	0.43	0.43
D-Lysine	1.11	1.06
D-Methionine	0.49	0.46
D-Methionine + cystine	0.84	0.79

D, digestible.

1) Premix provided per kg of diet: vitamin A, 8,500 IU; vitamin D_3_, 1500 IU; vitamin E, 25 IU; vitamin K, 2 mg; vitamin B_1_, 2.2 mg; vitamin B_2_, 8 mg; nicotinamide, 30 mg; vitamin B_5_, 35 mg; vitamin B_6_, 3.5 mg; vitamin B_12_, 0.01 mg; biotin, 0.18 mg; folic acids, 0.75 mg; Fe, 100 mg; Cu, 8.0 mg; Mn, 120 mg; Zn, 100 mg; I, 1.1 mg; Se, 0.3 mg.

**Table 2:** Primers for quantitative reverse-transcription polymerase chain reaction

Gene	Primer sequences (5' to 3')	Accession number	Product length (bp)
*GSS*	F: GCTCAGTGCCAGTTCCAGTT	XM_425692.4	115
	R: GGTCCCACAGTAAAGCCAAG		
*GSR*	F: CCAGAACACCACCAGAAAGG	XM_001235016.3	114
	R: TTACCAAAGAGCCGAAGTGC		
*CAT*	F:GTTTCCATCCTTCATCCATAGCC	NM_001031215.2	151
	R: GCGAAATCCATCAGGAATACCA		
*SOD1*	F: TTGTCTGATGGAGATCATGGCTTC	NM_205064.1	98
	R: TGCTTGCCTTCAGGATTAAAGTGAG		
*GPX1*	F: GCAAAGTGCTGCTGGTGGTCAA	NM_001277853.2	166
	R: ATCTCCT CGTTGGTGGCGTTCT		
*NRF2*	F: TGACCCAGTCTTCATTTCTGC	NM_205117.1	186
	R: GGGCTCGTGATTGTGCTTAC		
*ZO1*	F: GGTGCTTCCAGTGCCAACAGAA	XM_040680632.1	182
	R: GCCAACCGTAGACCATACTCTTCATT		
*Occludin*	F: GCAGATGTCCAGCGGTTACTACTAC	NM_205128.1	177
	R: GCGAAGAAGCAGATGAGGCAGAG		
*Claudin-1*	F: GGAGGATGACCAGGTGAAGAAGATG	NM_001013611.2	115
	R: CCGAGCCACTCTGTTGCCATAC		
*Claudin-2*	F: CGCTCACCCTCATTGGA	NM 001277622.1	124
	R: AACTCACTCTTGGGCTTCTG		
*β-actin*	F:CTGGTATTGTGATGGACTCTGGTGATG	NM_205518.1	162
	R: TGGTGGTGAAGCTGTAGCCTCTC		

*GSS*, glutathione synthetase; *GSR*, glutathione reductase; *CAT*, catalase; *SOD1*, superoxide dismutase 1; *GPX1*, glutathione peroxidase 1; *NRF2*, nuclear factor erythroid 2-related factor 2; *ZO1*, zonula occludens 1.

**Table 3:** Effects of dietary FA on growth performance of broilers.

Items	CON^1)^	FA100^1)^	FA200^1)^	SEM	p-value
Initial BW (g)	43.78	43.79	43.82	0.30	0.99
Starter phase (day 0 to day 2^1)^					
BW day 21 (g)	848.04^b^	885.95^a^	910.77^a^	9.23	<0.01
ADG (g)	38.29^b^	40.10^a^	41.28^a^	0.43	<0.01
ADFI (g)	52.23	52.72	53.98	0.73	0.24
F/G	1.36^a^	1.32^b^	1.31^b^	0.008	<0.01
Grower phase (day 21 to day 42)					
BW day 42 (g)	2,498.94^b^	2,528.28^b^	2,649.93^a^	28.66	<0.01
ADG (g)	78.61^b^	78.21^b^	82.82^a^	1.00	<0.01
ADFI (g)	138.43	138.10	140.11	0.99	0.32
F/G	1.76^a^	1.77^a^	1.69^b^	0.015	<0.01
Whole phase (day 0 to day 42)					
ADG (g)	58.46^b^	59.15^b^	62.05^a^	0.68	<0.01
ADFI (g)	95.33	95.41	97.05	0.83	0.28
F/G	1.63^a^	1.61^a^	1.56^b^	0.01	<0.01

FA, ferulic acid; SEM, standard error of the mean; BW, body weight; ADG, average daily gain; ADFI, average daily feed intake; F/G, feed to gain ratio (g:g).

1) CON, control group fed the basal diet; FA100, fed the basal diet supplemented with 100 mg/kg FA; FA200, fed the basal diet supplemented with 200 mg/kg FA.

a,b Different superscript letters within a row indicate significance (n = 8, p<0.05).

**Table 4:** Effects of dietary FA on serum parameters of intestinal permeability and antioxidant status of broilers

Items	CON^1)^	FA100^1)^	FA200^1)^	SEM	p-value
Day 21					
T-SOD (U/mL)	246.69	264.05	272.12	17.09	0.57
GSH-Px (U/mL)	473.77^b^	532.10^ab^	583.46^a^	29.11	0.049
CAT (U/mL)	1.29	1.32	1.35	0.057	0.77
GSH (mg/L)	4.41	4.79	4.68	0.27	0.59
MDA (nmol/mL)	3.11^a^	2.22^b^	1.95^b^	0.24	<0.01
DAO (U/L)	27.87^a^	21.21^b^	19.22^b^	1.85	<0.01
D-lactic acid (ng/mL)	6.21^a^	5.06^ab^	4.56^b^	0.39	0.02
Day 42					
T-SOD (U/mL)	355.90^b^	363.19^b^	426.48^a^	10.84	<0.01
GSH-Px (U/mL)	743.63^b^	795.39^ab^	851.41^a^	27.21	0.048
CAT (U/mL)	3.39	3.82	4.09	0.25	0.16
GSH (mg/L)	5.22	5.07	5.50	0.45	0.79
MDA (nmol/mL)	4.60^a^	4.31^ab^	3.54^b^	0.24	0.015
DAO (U/L)	21.35^a^	18.91^a^	14.32^b^	1.24	<0.01
D-lactic acid (ng/mL)	4.37	4.04	3.71	0.35	0.49

FA, ferulic acid; SEM, standard error of the mean; T-SOD, total superoxide dismutase; GSH-Px, glutathione peroxidase; CAT, catalase; GSH, reduced form of glutathione; MDA, malondialdehyde; DAO, diamine oxidase.

1) CON, control group fed the basal diet; FA100, fed the basal diet supplemented with 100 mg/kg FA; FA200, fed the basal diet supplemented with 200 mg/kg FA.

a,b Different superscript letters within a row indicate significance (n = 8, p<0.05).

**Table 5:** Effects of dietary FA on antioxidant capacity of intestinal mucosa in broilers on d 21

Items	CON^1)^	FA100^1)^	FA200^1)^	SEM	p-value
Duodenum					
T-SOD (U/mg protein)	235.23	239.66	241.45	9.26	0.89
GSH-Px (U/mg protein)	64.85	67.23	67.59	2.96	0.78
CAT (U/mg protein)	3.66	3.72	3.77	0.29	0.96
GSH (μg/mg protein)	9.56	10.01	10.41	0.54	0.55
MDA (nmol/ mg protein)	0.74	0.68	0.70	0.10	0.91
Jejunum					
T-SOD (U/mg protein)	206.75	232.70	231.68	8.32	0.07
GSH-Px (U/mg protein)	66.04	69.06	76.50	3.58	0.13
CAT (U/mg protein)	4.74	4.74	4.99	0.29	0.78
GSH (μg/mg protein)	9.36^b^	11.17^a^	11.66^a^	0.50	0.014
MDA (nmol/mg protein)	0.99^a^	0.69^b^	0.62^b^	0.08	0.049
Ileum					
T-SOD (U/mg protein)	334.88^b^	384.15^a^	390.47^a^	16.27	0.049
GSH-Px (U/mg protein)	106.89	116.69	117.29	16.29	0.26
CAT (U/mg protein)	3.86	3.53	4.45	0.39	0.25
GSH (μg/mg protein)	13.08^b^	15.91^a^	16.22^a^	0.72	0.02
MDA (nmol/ mg protein)	1.14^a^	0.76^b^	0.71^b^	0.09	<0.01

FA, ferulic acid; SEM, standard error of the mean; T-SOD, total superoxide dismutase; GSH-Px, glutathione peroxidase; CAT, catalase; GSH, reduced form of glutathione; MDA, malondialdehyde.

1) CON, control group fed the basal diet; FA100, fed the basal diet supplemented with 100 mg/kg FA; FA200, fed the basal diet supplemented with 200 mg/kg FA.

a,b Different superscript letters within a row indicate significance (n = 8, p<0.05).

**Table 6:** Effects of dietary FA on antioxidant capacity of intestinal mucosa in broilers on d 42

Items	CON^1)^	FA100^1)^	FA200^1)^	SEM	p-value
Duodenum					
T-SOD (U/mg protein)	258.99	257.84	265.42	9.18	0.82
GSH-Px (U/mg protein)	66.48	68.88	72.20	4.22	0.64
CAT (U/mg protein)	5.23	5.80	5.89	0.54	0.65
GSH (μg/mg protein)	10.66	11.00	11.69	0.65	0.54
MDA (nmol/mg protein)	1.02	0.92	0.77	0.19	0.68
Jejunum					
T-SOD (U/mg protein)	240.18^b^	262.12^ab^	301.69^a^	13.84	0.016
GSH-Px (U/mg protein)	86.40	90.15	100.22	4.46	0.10
CAT (U/mg protein)	4.47	4.09	4.82	0.51	0.60
GSH (μg/mg protein)	12.25	12.66	13.68	0.62	0.26
MDA (nmol/ mg protein)	1.16	1.08	0.78	0.17	0.29
Ileum					
T-SOD (U/mg protein)	308.81	315.70	337.98	9.76	0.11
GSH-Px (U/mg protein)	113.41^b^	125.83^ab^	143.45^a^	6.99	0.02
CAT (U/mg protein)	7.68	7.36	7.87	0.52	0.79
GSH (μg/mg protein)	13.77	14.02	15.34	0.72	0.28
MDA (nmol/mg protein)	1.21^a^	1.20^a^	0.98^b^	0.06	0.02

FA, ferulic acid; SEM, standard error of the mean; T-SOD, total superoxide dismutase; GSH-Px, glutathione peroxidase; CAT, catalase; GSH, reduced form of glutathione; MDA, malondialdehyde.

1) CON, control group fed the basal diet; FA100, fed the basal diet supplemented with 100 mg/kg FA; FA200, fed the basal diet supplemented with 200 mg/kg FA.

a,b Different superscript letters within a row indicate significance (n = 8, p<0.05).
